# Pre-Exposure Prophylaxis for the Prevention of HIV Infection in High Risk Populations: A Meta-Analysis of Randomized Controlled Trials

**DOI:** 10.1371/journal.pone.0087674

**Published:** 2014-02-03

**Authors:** Junjun Jiang, Xiaoyi Yang, Li Ye, Bo Zhou, Chuanyi Ning, Jiegang Huang, Bingyu Liang, Xiaoni Zhong, Ailong Huang, Renchuan Tao, Cunwei Cao, Hui Chen, Hao Liang

**Affiliations:** 1 Guangxi Key Laboratory of AIDS Prevention and Treatment, Guangxi Medical University, Nanning, Guangxi, China; 2 School of Public Health, Guangxi Medical University, Nanning, Guangxi, China; 3 School of Public Health, Chongqing Medical University, Chongqing, China; 4 Geriatrics Digestion Department of Internal Medicine, The First Affiliated Hospital of GuangXi Medical University, Nanning, Guangxi, China; Tulane University, United States of America

## Abstract

**Background:**

Nearly ten randomized controlled trials (RCTs) of pre-exposure prophylaxis (PrEP) have been completed or are ongoing worldwide to evaluate the effectiveness of PrEP in HIV transmission among HIV-uninfected high risk populations. The purpose of this study was to evaluate the role of PrEP to prevent HIV transmission through a Mata-analysis.

**Methods:**

A comprehensive computerized literature search was carried out in PubMed, EMbase, Ovid, Web of Science, Science Direct, Wan Fang, CNKI and related websites to collect relevant articles (from their establishment date to August 30, 2013). The search terms were “pre-exposure prophylaxis”, “high risk population”, “HIV infection”, “reduction”, “relative risk” and “efficacy”. We included any RCT assessing PrEP for the prevention of HIV infection in high risk populations. Interventions of the studies were continuously daily or intermittent doses of single or compound antiretrovirals (ARVs) before HIV exposure or during HIV exposure. A meta-analysis was conducted using Stata 10.0. A random-effects method was used to calculate the pooled relative risk (RR) and 95% confidence intervals (CI) for all studies included.

**Results:**

Seven RCTs involving 14,804 individuals in high risk populations were eligible for this study. The number of subjects in the experimental groups was 8,195, with HIV infection rate of 2.03%. The number of subjects in the control groups was 6,609, with HIV infection rate of 4.07%. The pooled RR was 0.53 (95% CI = 0.40∼0.71, *P*<0.001). The re-analyzed pooled RR were 0.61 (95% CI = 0.48∼0.77, *P*<0.001), 0.49 (95% CI = 0.38∼0.63, *P*<0.001), respectively, by excluding the largest study or two studies without statistical significance. Publication bias analysis revealed a symmetry funnel plot. The fail-safe number was 1,022.

**Conclusion:**

These results show that PrEP is an effective strategy for reducing new HIV infections in high risk populations.

## Introduction

Thirty years after HIV/AIDS was first identified as a serious disease, more than 60 million people have been infected with HIV and approximately 30 million people have died of AIDS. HIV remains a significant global health problem and a huge burden for our society. At the end of 2011, there were an estimated 34.0 (31.4∼35.9) million people living with HIV/AIDS globally, with 2.5 (2.2∼2.8) million new HIV infections [1]. The number of people infected with HIV will continue to increase unless effective interventions are established. Previous interventions to prevent HIV infection were largely dependent on male-controlled methods (male condoms, male circumcision and abstinence). However, more than 90% of all adolescent and adult HIV infections worldwide have resulted from heterosexual sex behaviors. Women are more vulnerable to heterosexual transmission of HIV due to substantial mucosal exposure to seminal fluids as well as social and biological factors [2], [3]. A series of unwilling interventions have highlighted the need for behavioral strategies to accompany biomedical strategies, especially for women to protect themselves against HIV infection. Therefore, female-controlled prevention has been proposed as a novel strategy to fill this gap.

Traditional interventions have been known to be poorly effective in HIV prevention. It is important to include new approaches to prevent HIV transmission, for example pre-exposure prophylaxis (PrEP). Recently, the prophylactic use of ARVs in preventing the sexual transmission of HIV, both orally and topically, has shown great promise [4], [5]. PrEP refers to the use of one or a combination of ARVs in HIV-negative individuals to prevent HIV infection [6]–[8]. In 2001, the nucleotide reverse transcriptase inhibitor tenofovir disoproxil fumarate (TDF) was approved for clinical therapy of HIV/AIDS. And then the combination of tenofovir and emtricitabine (TDF/FTC) was approved in 2004 [9]. In the absence of an effective vaccine at present, PrEP might be a reliable intervention to protect high risk HIV-negative people from HIV infection [10]–[13].

Animal studies have shown that daily or intermittent PrEP with TDF/FTC can exploit early viral vulnerabilities and effectively prevent HIV infection [14]–[16]. Mathematical models estimate that, over the next 10 years, an effective PrEP program could prevent 2.7∼3.2 million new HIV-1 infections in sub-Saharan Africa [17], [18]. Model simulations have also shown that an effective PrEP program could substantially reduce the incidence of HIV transmission in populations at high risk of infection [9], [19]. A review of clinical randomized controlled trials (RCTs) has indicated the important protective effects of oral antiretroviral drugs in the Cochrane Collaboration [20]. However, this review only included various types of oral PrEP in 9849 participants, and did not include studies involving topical application of ARVs (e.g., vaginal gels). Since then, RCTs have been initiated in multiple high risk populations with standard dosing of ARVs. However, there has been no meta-analysis of the role of PrEP in HIV prevention, both orally and topically. Therefore, this study evaluates the effectiveness of PrEP in preventing HIV infection in high risk populations through a meta-analysis.

## Methods

### Search methods

We included any RCT assessing antiretroviral drugs to prevent HIV infection in high risk populations. The search terms were “pre-exposure prophylaxis”, “high risk population”, “HIV infection”, “reduction”, “relative risk” and “efficacy”. A comprehensive computerized literature search was carried out in PubMed, EMbase, Ovid, Web of Science, Science Direct, Wan Fang (a Chinese bibliographic database), CNKI (China National Knowledge Infrastructure) and some other websites (ClinicalTrials.gov, hptn.org, Meta-Register) to collect the relevant literature (from establishment to August 2013).

### Inclusion criteria

Articles obtained from these searches and relevant references cited in the articles were screened and assessed independently by two reviewers for eligibility. Inclusion criteria were applied to all relevant RCTs as follows: (1) only RCTs evaluating the efficacy of PrEP on HIV infection; (2) subjects were high risk HIV-negative people; (3) interventions were daily or intermittent PrEP of single or combined ARVs before HIV exposure or during HIV exposure, both orally and topically; (4) the primary endpoint was new HIV infections.

### Exclusion criteria

Studies were excluded if the number of the subjects and events in the experimental group and control group were not well-described. Duplicate publications were also excluded.

### Data Extraction

Two reviewers extracted the data of all included trials using a standardized form. Data extracted were as follows: (1) author and publication year; (2) phase of the trial; (3) location and population; (4) enrolment date and follow-up year; (5) interventions; (6) number of participants; (7) endpoints; (8) dropout number in each trial; (9) protection rate or relative risk (RR). If there was different information between two reviewers, the third reviewer would participate to determine the correct information. Additionally, if the data were not sufficient for the meta-analysis, telephone or email was used to get more information.

### Assessment of study quality

The methodological quality of the included RCTs was assessed using the standard Jadad score based on the adequacy of randomization, blinding and follow up, with a maximum score of 5 points. A score of 0∼2 indicates low quality, while a score of 3∼5 indicates high quality [21].

### Data synthesis and meta-analysis

Data were analyzed using Stata10.0 software (American Computer Resource Center). We calculated the relative risk (RR) with 95% confidence intervals (CI). Statistical heterogeneity was quantified using the I^2^ statistic to measure the proportion of the overall variation and assessed for strength of the evidence using the chi-squared test [22]. In pooling the data from these included trials, a fixed-effects model was applied using the method of Mantel-Haenzel (M-H) when there was no statistically significant heterogeneity. A random-effects model was employed using the method of DerSimonian and Laird (D+L) if statistically significant heterogeneity was detected. Statistical significance of the test for heterogeneity was set at 0.05. Sensitivity analysis was performed by re-analysis, excluding one low quality study and one study with no statistical significance. A funnel plot was applied to examine the potential publication bias in the meta-analysis. The fail-safe number was calculated as [
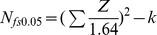
] [23].

## Results

### Search results

**Figure 1:**
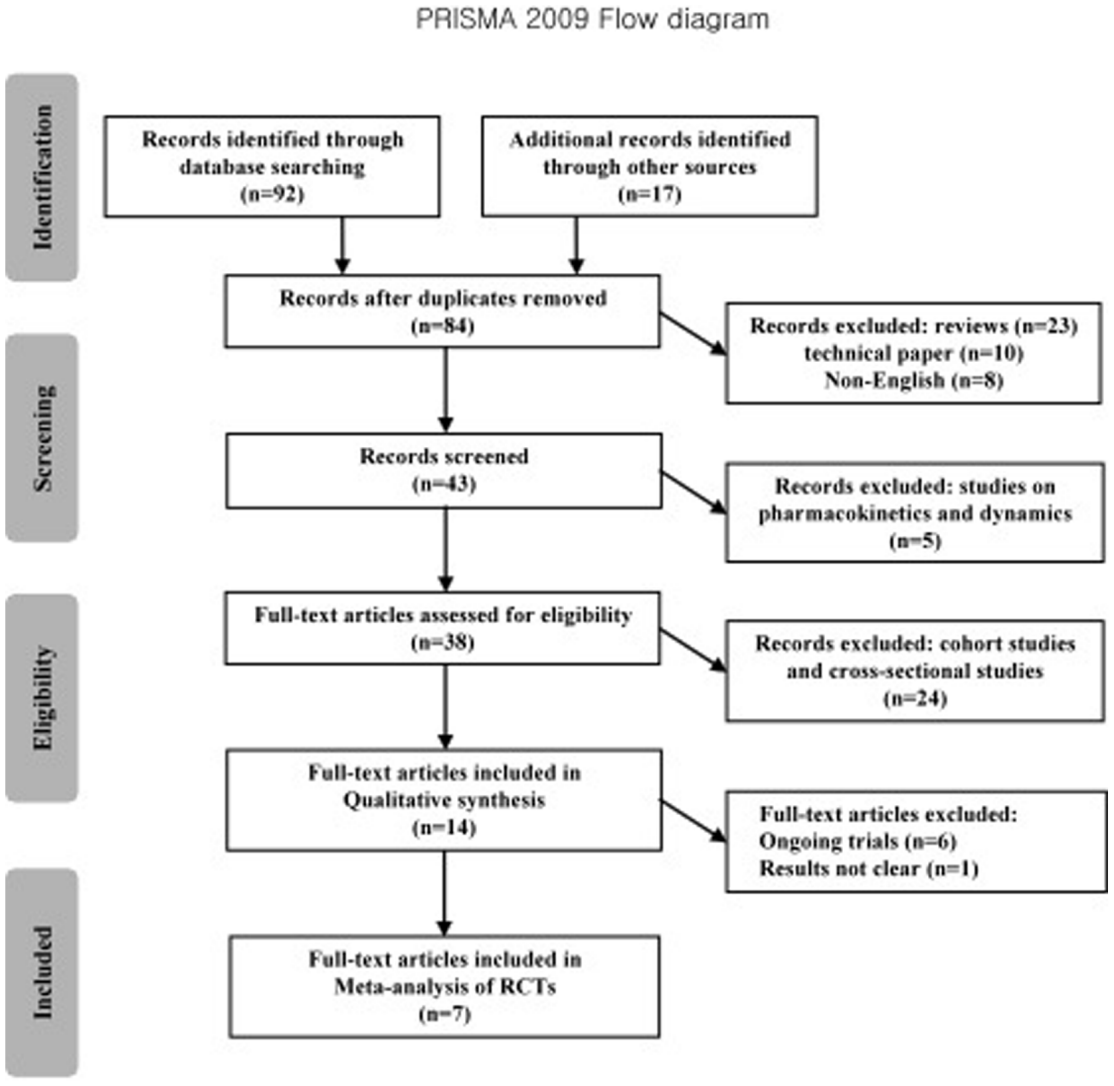
Literature search flow chart.

The detailed search step is summarized in the flow chart (Figure 1). We initially searched 109 citations. After screening and exclusion, thirty-eight articles were further scrutinized. Finally, we identified seven RCTs that meet the inclusion criteria [24]–[30], including one that was terminated early [29]. Four of the studies [24], [26], [27], [29] were multinational trials. The study of Peterson et al. was conducted in Garna, Cameroon and Nigeria among 936 sexually active women [27]. The study of Grant et al. was conducted in Peru, South Africa, Brazil, Thailand, United States and Ecuador, including 2,499 men who have sex with men (MSM) [26]. The study of Baeren et al. was conducted in Kenya and Uganda among 4,747 HIV-discordant heterosexual couples [24]. The study of Van Damme et al. was conducted in Kenya, South Africa and Tanzania among 2,120 women [29]. The study of Choopanya et al. enrolled 2,413 injecting drug users (IDUs) from drug-treatment clinics in Bangkok, Thailand [30]. The other ten articles were excluded because they are still on-going, duplicate publications, or contain no details about the outcomes [31]–[40].

### Description of included RCTs

Table 1 shows the key characteristics of the included trials. All trials used TDF-containing products, either TDF alone or in combination with FTC. Seven studies were placebo-controlled and one had two intervention groups. Most of the participants lived in highly epidemic HIV areas, such as South Africa, Botswana, Kenya and Uganda. The included trials were reported between 2007 and 2013, and their sample sizes ranged from 889 to 4,747 participants. All 14,804 participants were HIV negative individuals in a high infection risk. Ages ranged from 18∼67 years. All the participants provided written informed consent, received HIV testing and were provided with comprehensive HIV prevention services (HIV pre- and post-test counseling, HIV risk reduction counseling, condoms and STI treatment). At the endpoint, five studies showed that PrEP was effective, while the other two did not. The study of Peterson et al. with daily oral 300 mg TDF in Ghana, Nigeria and Cameroon showed no differences in adverse events or grade 3 or 4 laboratory abnormalities between placebo and TDF users [27]. There were fewer infections in the TDF group (two events versus six events in the placebo group), even though the study was not of sufficient size or duration to examine the efficacy of tenofovir. The study of Van Damme et al. with daily oral TDF/FTC assigned to 2,120 HIV negative women in Kenya, South Africa and Tanzania did not significantly reduce the rate of HIV infection, as compared with the placebo group [29]. HIV infections occurred in thirty-three women in the TDF/FTC group and in thirty-five in the placebo group. The study was stopped early, because of lack of efficacy.

**Table 1:** Characteristics of the included trials.

Study, year	Phase	Location	Population	Started year (follow-up)	Intervention	Number: infection/total (Experimental)	Number: infection/total (Control)	Protection rate
Peterson et al., 2007 [27]	phase II	Garna, Nigeria, Cameroon	936, sexually active women	2004 (1 y)	Daily oral 300 mg TDF	2/469	6/467	Protection rate = 65%, *P* = 0.24
Abdool et al., 2010 [25]	phase IIB	South Africa	889, sexually active women	2007 (mean, 1.5 y)	40 mg TDF vaginal gel (BAT24)*	38/445	60/444	Protection rate = 39%, (95%CI:6∼60%), *P* = 0.017
Grant et al., 2010 [26]	phase III	Peru, South Africa, Brazil, Thailand, United States, Ecuador	2499, MSM	2007 (median, 1.2 y; maximum, 2.8 y)	Daily oral FTC/TDF (200 mg FTC+300 mg TDF)	36/1251	64/1248	Protection rate = 44%, (95%CI:15∼63%), *P* = 0.005
Baeten et al., 2011 [24]	phase III	Kenya, Uganda	4747, HIV-discordant heterosexual couples	2008 (3 y)	Daily oral TDF or FTC/TDF (FTC: 200 mg, TDF: 300 mg)	TDF:18/1584; FTC/TDF:13/1579	47/1584	TDF protection rate = 62% (95%CI: 34∼78%), *P* = 0.0003; FTC/TDF protection rate = 73% (95%CI: 49∼85%), *P*<0.0001
Van Damme et al., 2012 [29]	phase III	Kenya, South Africa, Tanzania	2120, women	2009 (1 y)	Daily oral FTC/TDF (N/A)	33/1062	35/1058	Protection rate = 6%, *P* = 0.81
Thigpen et al., 2011 [28]	phase II	Botswana	1200, heterosexual men and women	2007, (median, 1.1 y; maximum, 3.7 y)	Daily oral FTC/TDF (N/A)	9/601	24/599	Protection rate = 62.6%, (95%CI:21.5∼83.4%), *P* = 0.003
Choopanya et al., 2013 [30]	phase III	Bangkok, Thailand	2413, IDUs	2005, (median, 4.0 y; maximum, 6.9 y)	Daily oral 300 mg TDF	17/1204	33/1209	Protection rate = 48.9%, (95%CI: 9.6∼72.2%), *P* = 0.01

Note: MSM, men who have sex with men; IDUs, injecting drug users; FTC, emtricitabine; TDF, tenofovir disoproxil fumarate; PR, Protection Rate; CI, 95% confidence intervals; BAT24, one dose of tenofovir gel within 12 hours before sex and a second dose within 12 hours after sex; N/A, not available.

### Methodological Quality Assessment

The quality of the included studies is shown in Table 2. All trials were prospective, randomized, double-blinded and placebo-controlled, and were received Jadad scores of 3 (n = 1), 4 (n = 1) or 5 (n = 5) points. All the studies described the baselines in both the experimental and the control groups, and they were similar in important demographic respects. According to the Jadad score, all the studies can be considered high quality research, with scores ≥3.

**Table 2:** Quality assessment of the included trials.

Study, year	Randomization	Blinding	Placebo-controlled	Dropout (n)	Jadad score
Peterson et al., 2007	adequate	double-blind	yes	162	5
Abdool et al., 2010	unclear	double-blind	yes	6	4
Grant et al., 2010	adequate	double-blind	yes	48	5
Baeten et al., 2011	unclear	double-blind	yes	N/A	3
Van Damme et al., 2012	adequate	double-blind	yes	266	5
Thigpen et al., 2012	adequate	double-blind	yes	115	5
Choopanya et al., 2013	adequate	double-blind	yes	355	5

Note: Adequate if the allocation sequence was generated by a computer or random number table. Unclear if the trial was described as randomized, but the method used for the allocation sequence generation was not described; N/A, not available.

### Meta-analysis

**Figure 2:**
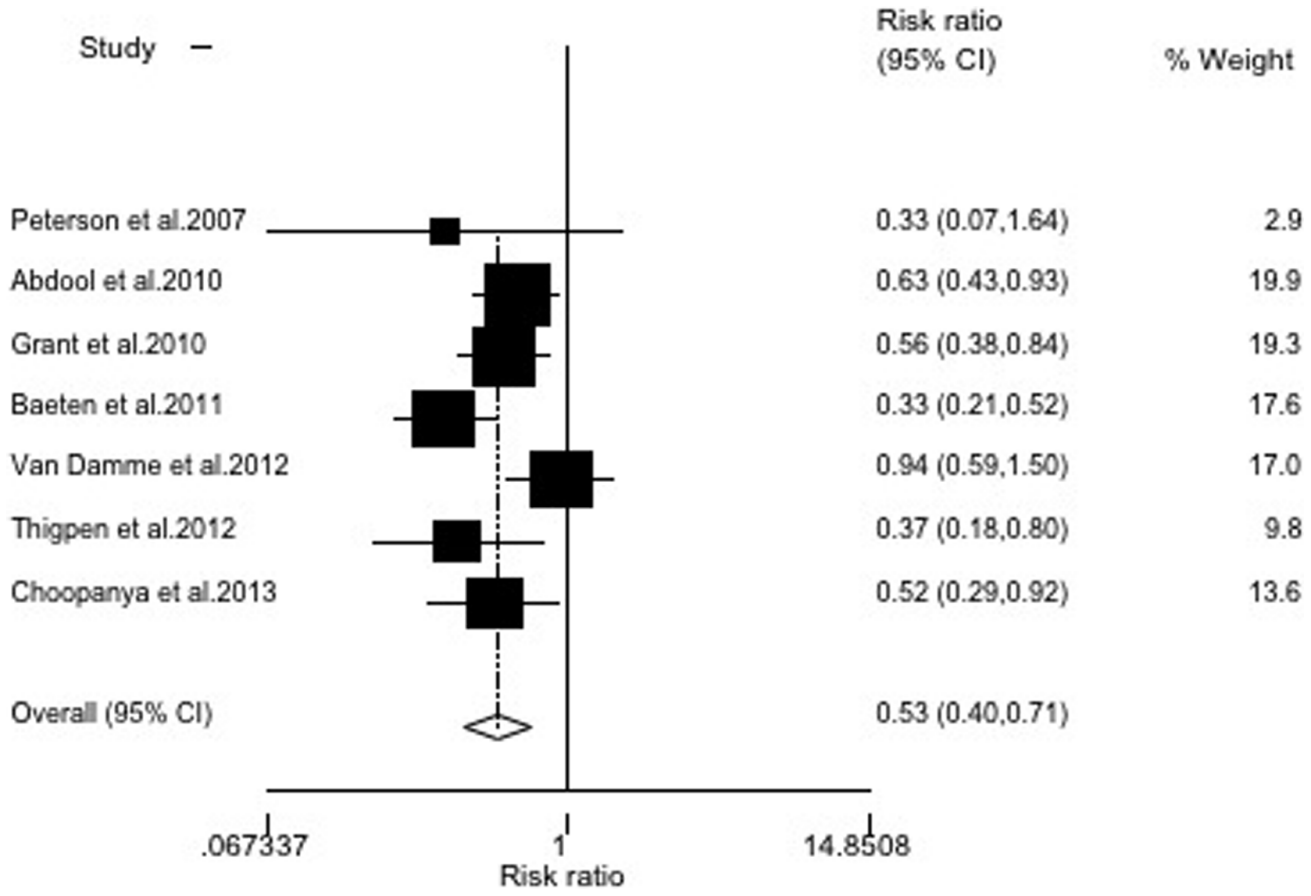
Random-effects model meta-analysis. Heterogeneity chi-squared = 11.91 (d.f. = 6), *P* = 0.064; test of RR = 1∶z = 4.29, *P*<0.001.

Seven papers describing RCTs were enrolled, including 14,804 subjects in high risk populations. The number of experimental subjects was 8,195, with HIV infection rate of 2.03%. The number of control subjects was 6,609, with HIV infection rate of 4.07%. The result of the heterogeneity test (X^2^ = 11.91, *P* = 0.06<0.1, I^2^ = 50%) showed that there was heterogeneity among these studies. We therefore chose the random-effects model for the meta-analysis. It showed that the pooled relative risk (RR) was 0.53 (95% CI = 0.40∼0.71, *P*<0.001) (Figure 2).

### Sensitivity analysis

**Figure 3:**
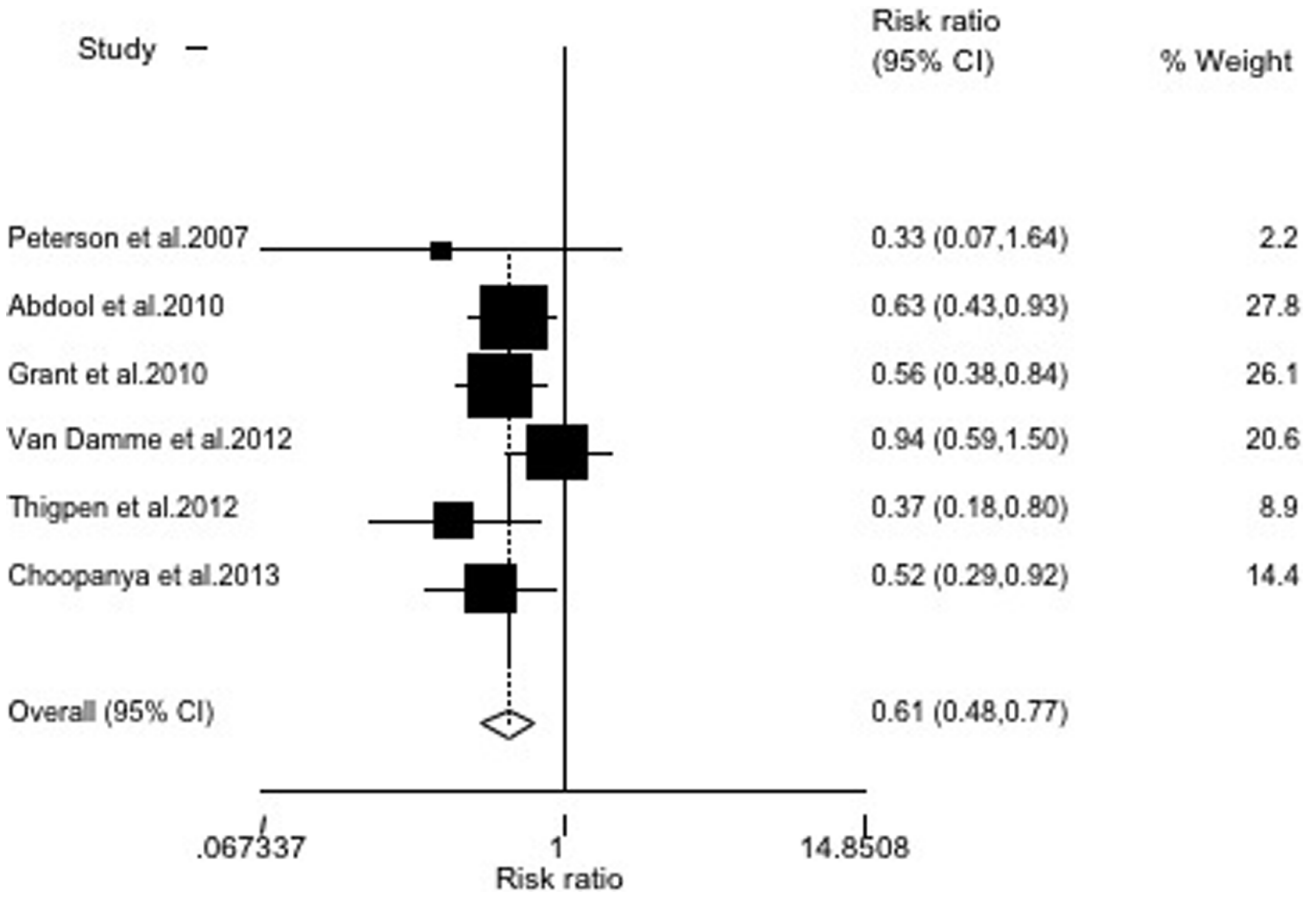
Random-effects model meta-analysis excluding the largest study. Heterogeneity chi-squared = 5.95 (d.f. = 5), *P* = 0.311; test of RR = 1∶z = 4.12, *P*<0.001.

**Figure 4:**
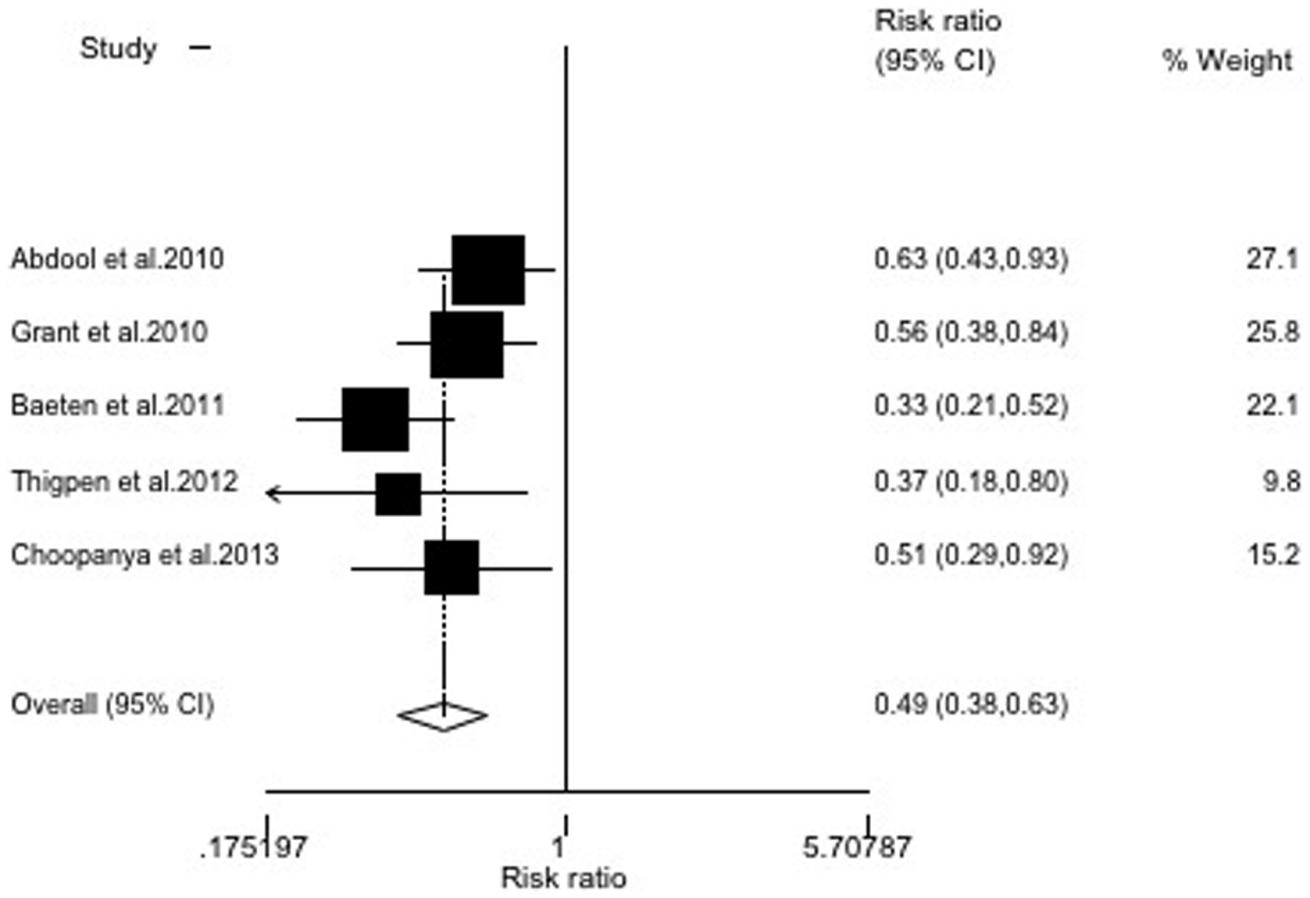
Random-effects model meta-analysis, excluding two studies with no statistical significance. Heterogeneity chi-squared = 5.58(d.f. = 4), *P* = 0.233; test of RR = 1∶z = 5.51, *P*<0.001.

There was wide variation in the sample size, the largest study consisted of 4,747 participants (32.1% of the total subjects included in the meta-analysis) and the smallest study consisted of 889 participants. To examine the influence of the largest study on the meta-analysis, we re-analyzed the data by excluding the largest study (which was low in quality). The resulting RR was 0.61 (95% CI = 0.48∼0.77), indicating that this study did not largely influence the meta-analysis (Figure 3). In addition, when we excluded the Peterson and Van Damme studies, which had no statistical significance, the RR was 0.49 (95% CI = 0.38∼0.63)(Figure 4). The remaining studies, after excluding the largest study and two without statistical significance, produced results similar to the overall meta-analysis.

### Publication bias

**Figure 5:**
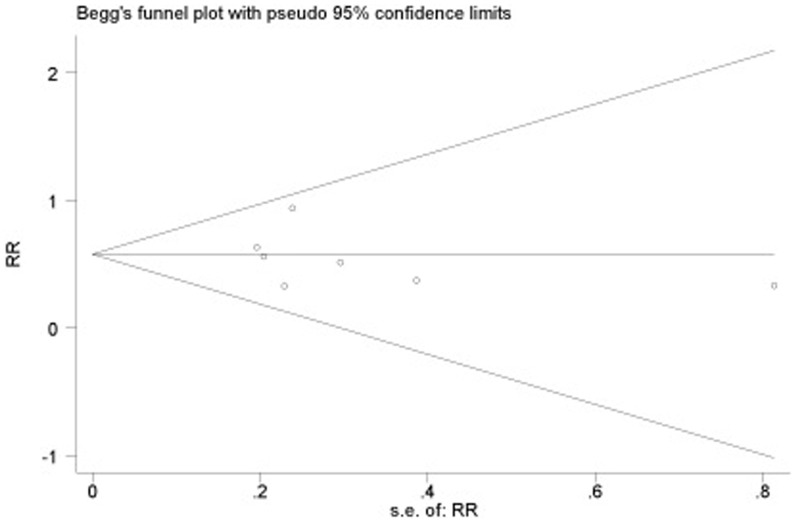
Funnel plot for the publication bias of all seven included trials.

A funnel plot of the data is presented in Figure 5. The included studies appear in the funnel plot completely and are distributed around the pooled RR, with large sample size results at the top. Meantime, we performed funnel chart linear regression model analysis. The result showed that the intercept's 95% CI = −3.14∼1.93 contained 0 (*P* = 0.565>0.1), indicating that the funnel plot was symmetrical. The fail-safe number of this study was 1,022, which means that it would need at least 1,022 unpublished, especially negative results in the literature to override the conclusion of the meta-analysis.

## Discussion

This meta-analysis provides evidence that PrEP is associated with a reduced risk of HIV infection in high risk populations. The strongest association was seen in the Thigpen study, with a protective rate of 62.6% in HIV-uninfected, sexually active, healthy males and females. As a new prevention method, antiretroviral drugs effectively prevent HIV transmission at birth, during breastfeeding and after occupational exposure [41], [42]. Proof-of-concept that PrEP protects against sexual HIV acquisition has been demonstrated in clinical trials. Nowadays, PrEP is still in the clinical trial phases, and large phase III clinical randomized controlled trials are ongoing.

There are several strengths and limitations to consider in our analysis and in the included trials. The strengths of our meta-analysis include two extensive studies (with a sample size more than two thousand subjects), thirteen different sites (Garna, Cameroon, Nigeria, South Africa, Peru, Brazil, Thailand, United States, Ecuador, Botswana, Kenya, Uganda and Tanzania) and different HIV high risk populations (MSM, IDUs, HIV-discordant heterosexual couples and heterosexual men and women).

Perhaps the most important limitation of our meta-analysis is the small number of studies available to fully explore how PrEP prevents the acquisition of HIV infection in high risk populations. The analysis of these RCTs was restricted to a part of high risk populations. Other ongoing clinical trials on oral or topical HIV PrEP including other high risk populations have no results reported yet [43].

The second limitation to the result was that two studies were stopped early for some reasons. One phase II safety study in Ghana, Nigeria and Cameroon among 936 female sex workers showed no difference in the frequency of adverse events between those taking tenofovir and placebo. The initial results of this study did not show a good prophylactic effect, and this trial was not completed as planned. Two sites (Nigeria and Cameroon) were closed either before the planned number of participants had been recruited or before all participants had completed full follow-up. Therefore, this study did not have sufficient power to assess the differences between trial arms in the primary efficacy analysis. Moreover, the study of Van Damme et al. showed that prophylaxis with TDF/FTC did not significantly reduce the rate of HIV infection. It was stopped early, because of lack of efficacy, and with 13% participants lost to follow-up.

The third limitation of the literature was the different formulations and dosages of antiretroviral drugs in the included trials. In Peterson and Choopanya studies, participants were randomized to once daily use of 300 mg of TDF or placebo. In Grant, Van Damme and Thigpen studies, participants were randomized to once daily use of FTC/TDF (200 mg FTC+300 mg TDF) or placebo. In study of Abdool et al., women applied one dose of tenofovir gel within 12 hours before sex and a second dose of tenofovir gel as soon as possible within 12 hours after sex, but no more than two doses of tenofovir gel in a 24-hour period. In study of Baeten et al., participants were randomized to a three-arm trial of once daily use of TDF, FTC/TDF or placebo.

The fourth limitation was that there are several areas that require further research and on-going surveillance if PrEP is to become part of an HIV prevention program. It is impossible for PrEP to provide 100% protection against HIV, and some people will become infected while using PrEP. Furthermore, this may help the virus to mutate, resulting in restricted future treatment options. Moreover, informal drug sharing, black-market use or imperfect screening might result in some people who are HIV-positive inadvertently taking prophylaxis.

The final limitation was that only articles in English were included, so there might be a language bias.

## Conclusion

The available relevant studies were included in our meta-analysis. Our findings support that PrEP has protective effect against HIV infection in high risk populations. If other on-going and large scale studies provide more data on the relationship between PrEP and HIV infection in coming years, it will help to further define the role of PrEP in the prevention of HIV transmission. However, as a strategy, PrEP should always be regarded as a component of prevention but not a replacement for existing methods, and should be integrated as much as possible into existing programs to bring us closer to our goal of full prevention.

## Supporting Information

Table S1 PRISMA Checklist
(DOC)
